# Visualization of super-selective Wada with computed tomography perfusion: toward a novel option in preoperative functional mapping

**DOI:** 10.3389/fnimg.2026.1876584

**Published:** 2026-10-26

**Authors:** Shunsuke Nakae, Motoharu Hayakawa, Masanobu Kumon, Noeru Kawase, Kenichiro Suyama, Eiji Fujiwara, Daijiro Kojima, Shigeo Ohba, Shogo Imamura, Yuriko Sato, Yoko Inamoto, Masaki Fukunaga, Yuichi Hirose

**Affiliations:** 1 Department of Neurosurgery, Fujita Health University, Toyoake, Aichi, Japan; 2 Department of Rehabilitation, Fujita Health University Hospital, Toyoake, Aichi, Japan; 3 Faculty of Rehabilitation, School of Health Sciences, Fujita Health University, Toyoake, Aichi, Japan; 4 Division of Cerebral Integration, National Institute for Physiological Sciences, Okazaki, Aichi, Japan

**Keywords:** brain tumor, computed tomography perfusion, epilepsy surgery, functional mapping, seizure-onset zone, stereo-electroencephalography, super-selective Wada

## Abstract

**Introduction:**

The Wada test has long been a well-established method for evaluating hemispheric dominance and functional localization; however, it has not been established as a visualizable functional imaging modality. Recently, the super-selective Wada test (ssWada) has emerged as a technique enabling more localized functional evaluation. By selectively catheterizing distal cerebral arterial branches, ssWada enables territory-based functional evaluation with higher spatial resolution than conventional approaches. In this study, we combined ssWada with computed tomography perfusion (CTP) to visualize pharmacologically inactivated brain regions preoperatively. These data were further fused with magnetic resonance imaging (MRI) and CT to propose a novel functional mapping approach delineating the relationship between perfusion-defined functional territories, lesions, and seizure onset zone (SOZ)-related stereoelectroencephalography (SEEG) contacts.

**Methods:**

Patients who underwent ssWada for preoperative evaluation of brain tumors or drug-resistant epilepsy at our institution were included. During ssWada, propofol was selectively injected via a microcatheter into the ICA and distal branches of the middle cerebral artery (MCA), as well as the anterior or posterior cerebral artery when necessary. Perfusion territories were simultaneously visualized using CTP. These datasets were subsequently fused with MRI and post-SEEG CT to evaluate the spatial relationship between perfusion-defined functional territories, lesions, and SOZ-related SEEG contacts.

**Results:**

Among the 23 patients, hemispheric dominance was left in 18 patients (78.3%), bilateral in 3 (13.0%), and right in 2 (8.7%). No complications were observed. In the MCA superior trunk territory, repetition, naming, and reading tasks showed positive rates of approximately 70%. In contrast, in the inferior trunk territory, repetition and reading demonstrated high positive rates (93.8%), whereas naming positivity was lower (56.3%). Fusion with MRI and post-SEEG CT enabled spatial visualization of lesions and SOZ-related SEEG contacts within perfusion-defined functional territories, facilitating individualized functional-anatomical assessment for surgical planning.

**Discussion:**

ssWada combined with CTP enables territory-based visualization of pharmacologically inactivated brain regions and provides a novel preoperative functional mapping approach with high spatial resolution. Fusion with MRI and post-SEEG CT further facilitates individualized functional-anatomical assessment of lesions and SOZ-related networks. These findings support the clinical utility of ssWada-CTP fusion imaging as a novel strategy for individualized preoperative functional brain mapping.

## 1 Introduction

Stereo-electroencephalography (SEEG) is the primary modality for localizing seizure-onset zones (SOZ) in focal epilepsy and enables three-dimensional assessment of epileptic activity, including deep structures (Gonzalez-Martinez et al., 2013; Bartolomei et al., 2017); however, functional evaluation of deep SEEG contacts remains challenging. When resection is considered, accurate functional characterization of SOZ-related SEEG contacts is essential for safe surgical planning and functional preservation. SEEG offers limited stimulation-based functional mapping, leaving reliable preoperative visualization of deep functional anatomy unresolved.

Various brain-mapping methods have been employed to maximize resection while preserving function in epilepsy and intra-axial tumors. Direct cortical stimulation during awake craniotomy remains the most reliable method and is considered the gold standard in glioma surgery (De Witt Hamer et al., 2012; Ius et al., 2021). After SEEG, assessing the function of SOZ-related contacts helps determine the indication for awake mapping. Noninvasive approaches such as functional magnetic resonance imaging (fMRI) and magnetoencephalography (MEG) are widely used; however, those strategies still struggle to balance reliability with spatial resolution (Silva et al., 2017; Papanicolaou et al., 1999; Cargnelutti and Tomasino, 2023).

The Wada test provides transient hemispheric inactivation and has long been used to assess language and memory lateralization (Wada, 1949; Wada and Rasmussen, 1960; Milner et al., 1962; Conradi et al., 2021). Super-selective Wada testing (ssWada) further improves spatial resolution by administering anesthetic agents at specific arterial branches (Kakinuma et al., 2022). Although reports on ssWada have increased (Kakinuma et al., 2022; Kakinuma et al., 2024; Kuruvilla et al., 2024), to our knowledge few studies have applied the Wada test as a preoperative functional mapping tool integrated into surgical planning.

In this study, we performed ssWada in an angiography suite equipped with computed tomography perfusion (CTP) to visualize brain regions perfused by propofol. These perfusion maps were coregistered with MRI and post-SEEG CT to identify functional territories associated with epileptogenic contacts, including those within cerebral cavernous malformations (CCM) and SOZ. We used this information to guide decisions regarding the need for awake mapping and the selection of intraoperative task design. We propose a preoperative functional-mapping approach that offers high spatial resolution and practical clinical utility, and we evaluate its feasibility and relevance in epilepsy surgery.

## 2 Materials and methods

### 2.1 Patients

We included 23 consecutive patients evaluated at Fujita Health University Hospital between August 2021 and June 2025 for surgical treatment of either brain tumors or drug-resistant epilepsy. Inclusion criteria were: (i) cases in which preoperative determination of language dominance was considered important, or (ii) cases in which assessment of a specific functional domain was required because the patient requested preservation of that function. These criteria encompassed left-handed patients or individuals with a history of early left-handedness followed by handedness switching; right-handed patients with an uncertain history of handedness conversion or those who requested confirmation of language dominance, often to potentially avoid awake craniotomy; and patients who wished to preserve a specialized functional domain whose cortical representation was unclear. All 23 consecutive patients meeting the inclusion criteria were included, with no exclusions. Because tasks were selected according to each patient’s clinical needs, tasks not performed because they were not clinically indicated were not considered missing data. Positive rates were calculated at the task level, using the total number of performed tasks as the denominator. Sex was defined as female or male as recorded in the medical records. The study was approved by the institutional review board of Fujita Health University Hospital. We used the STROBE reporting guideline to draft this manuscript.

### 2.2 Super selective Wada test

All procedures were performed in an angiography suite equipped with a 320-detector row CT scanner. The system included a 320-row CT scanner (Aquilion ONE; Canon Medical Systems, Tochigi, Japan) and a biplane angiography unit (Alphenix Sky+; Canon Medical Systems). All catheterizations were performed by board-certified neuroendovascular specialists of the Japanese Society of Neuroendovascular Therapy (JSNET). Under fluoroscopic guidance and local anesthesia of the right groin, a 5-Fr sheath was inserted, followed by placement of a 5-Fr guiding catheter (Envoy 5F, Cerenovus, Irvine, CA, USA) into the internal carotid artery (ICA) on the side to be tested.

For each ICA, a conventional Wada test was initially performed to confirm hemispheric language dominance. After selection of a unilateral ICA, patients were instructed to raise both arms and count aloud with the examiner. Propofol (1%, 10 mg/mL) was used for the injections. For injections from the guiding catheter, propofol was diluted tenfold with saline to a final concentration of 1 mg/mL and administered at a rate of 1 mL per count. Injection was stopped immediately upon the appearance of contralateral paresis, after which an additional 1 mL was administered before task testing. The off-label use of propofol for super-selective Wada testing was reviewed and approved by the institutional regulatory and ethics committees.

A microcatheter (Excelsior SL-10, Stryker, Kalamazoo, MI, USA) was advanced using a microguidewire (Synchro Select, Stryker) into branches of the middle cerebral artery or other relevant cerebral arteries based on lesion location identified on MRI, followed by CT perfusion (CTP) imaging. CTP parameters were as follows: tube voltage, 80 kV; tube current, 80 mA; rotation time, 1.0 s; and intermittent acquisition. Following the start of acquisition, 0.5 mL of contrast was injected over 2 s, and images were obtained for 15 s. Perfusion data were analyzed using block singular value deconvolution (bSVD), which is robust to contrast arrival delays and provides reliable perfusion estimates even in cases of arterial stenosis or collateral flow (Sasaki et al., 2009). Perfusion territories were derived from cerebral blood volume (CBV) maps. For super-selective injections through a microcatheter, undiluted propofol was injected at a rate of 0.1 mL per count; injection was terminated if paresis occurred, otherwise the full dose (10 mg) was delivered before functional testing. Thus, approximately 1 mg of propofol was delivered per count in every condition.

### 2.3 Neurocognitive assessment

Functional mapping was performed after intra-arterial propofol injection into the ICA and super-selectively catheterized cerebral arteries. Tasks assessed motor, language, calculation, and spatial attention functions. Language testing included repetition, naming, and reading, each administered in three trials. Repetition tasks consisted of single words and sentences. Reading tasks included words and/or sentences presented in hiragana and/or kanji. Calculation was assessed using single-digit addition. Spatial neglect was assessed using a line bisection test, with additional tasks added when clinically indicated.

Tasks were administered in a fixed order without pretest rehearsal by a certified speech-language-hearing therapist using standardized evaluation sheets, and all assessments were completed within 3 min of propofol injection.

Language impairment was defined as clear and sustained incorrect responses, response cessation, or marked delay relative to baseline. Dysarthria was defined as impaired articulation with preserved linguistic structure, whereas aphasia was defined as disruption of linguistic processing such as impaired word retrieval, repetition, or reading. Functional assessments were evaluated by two speech-language-hearing therapists and three neurosurgeons, who were aware of the injected arterial territory. When interpretation was uncertain, the recorded assessment was reviewed by an independent blinded evaluator, and the final classification was determined by consensus. For the line bisection test, a 20.5-cm horizontal line was used, and deviations >1.4 cm from the true center were considered abnormal, based on previously reported criteria using lines of approximately 20 cm in length (Pandian et al., 2014). Categorical data were summarized descriptively as frequencies and percentages. Given the exploratory nature of this study, the limited sample size, and the presence of multiple assessments within individual patients, no formal hypothesis testing was performed. Sex-based analyses were not performed in this study.

### 2.4 Radiological studies

CTP was performed using the angiography-suite 320-row CT scanner as described above. MRI studies were conducted at the Radiology Department of Fujita Health University Hospital. For image fusion, three-dimensional fluid-attenuated inversion recovery (3D FLAIR) sequences with fat suppression were used (field of view, 240 × 240 mm; acquisition matrix, 240 × 240; slice thickness, 0.75 mm; TR, 5000 ms; TE, 504.7 ms; TI, 1550 ms).

CTP-derived perfusion maps were manually coregistered to cortical and basal surfaces of MRI using a dedicated workstation (StealthStation S8 Surgical Navigation System, Medtronic, Minneapolis, MN, USA) to visualize spatial relationships between perfusion territories and functional deficits. In patients who underwent SEEG for drug-resistant focal epilepsy, postoperative non-contrast CT following depth-electrode implantation was additionally fused to evaluate anatomical relationships among perfusion territories, functional effects, and SOZ contacts. Patients were monitored for catheter-related complications throughout hospitalization. Radiation dose indices were retrospectively collected from available dose reports.

## 3 Results

### 3.1 Patients

The clinical characteristics of the 23 consecutive patients who underwent ssWada testing as part of presurgical evaluation for brain tumors or drug-resistant epilepsy are summarized in Table 1. The mean age was 43.6 ± 16.4 years, and 9 patients (39.1%) were female. Thirteen patients (56.5%) were left-handed, including those with a history of handedness conversion. Eighteen patients had brain tumors, most commonly gliomas, and epilepsy was present in 15 patients. Other structural lesions included CCM, intracranial hemorrhage, and focal cortical dysplasia (Table 1). No complications related to ssWada were observed. Language dominance was left in 18 patients (78.3%), bilateral in 3 (13.0%), and right in 2 (8.7%). No catheter-related complications were observed during hospitalization. Radiation dose data were available for 20 patients, with a cumulative dose-length product of 3,929 mGy·cm and a cumulative volume CT dose index of 239 mGy.

**Table 1:** Patient characteristics and vessels selected for super-selective Wada test.

No.	Age	Sex	Hand	Diagnosis	Lesion	Location of lesions	Dominant	SSW side	Selected vessels
1	37	M	L	Epilepsy	R	Parieto-occipital, temporal	L	L	MCA (2) + ACA
2	33	M	L	CCM, epilepsy	Bi	Multiple ccms	L	L	MCA (2) +ACA
3	50	M	L	Tumor, epilepsy	Bi	l temporal, bi thalami	L	L	MCA (2) +ACA
4	40	M	R	Tumor, epilepsy	L	Insula, frontal	L	L	MCA (2) +ACA
5	44	M	L	Tumor, epilepsy	L	Frontal	L	Bi	MCA (2) +ACA/MCA (2) +ACA
6	62	F	L	Tumor, epilepsy	R	Temporal	Bi	R	MCA (2) +PCA
7	18	M	R	Tumor, epilepsy	R	Temporal	L	R	MCA (2) +ACA
8	55	F	R	ICH, epilepsy	L	Parietal, temporal	L	L	MCA (3)
9	64	F	L	Tumor	R	Temporal	L	R	MCA (2)
10	39	M	R	Tumor, epilepsy	L	Temporal, hypothalamus	R	L	MCA (2)
11	17	M	L	Tumor, epilepsy	R	Temporal	Bi	R	MCA (2)
12	45	F	R	Tumor	R	Frontal	L	R	MCA (5)
13	24	M	R	Tumor	L	Temporal, insula	L	L	MCA (2)
14	44	F	L	Tumor	R	Frontal	L	R	MCA (2)
15	54	M	L	Tumors, epilepsy	Bi	Frontal	L	L	MCA (2) +ACA
16	74	F	R	CCM, epilepsy	R	Temporal	L	R	MCA (2) +ACA
17	17	F	R	Tumor	L	Temporal	R	Bi	MCA (2)/ MCA (2)
18	44	M	L	Tumor	R	Frontal	R	R	MCA (3)
19	71	M	L	Tumor	L	Parietal	Bi	L	MCA (2) +ACA
20	42	M	L	Tumor	R	Temporal	L	R	MCA (3)
21	36	F	R	FCD, epilepsy	L	Temporal	L	L	MCA (2)
22	30	F	R	Tumor, epilepsy	L	Parietal	L	L	MCA (3)
23	62	M	L	Tumor, epilepsy	L	Temporal	L	L	MCA (3)

This table summarizes the age, sex, handedness, underlying conditions, lesion laterality, language-dominant hemisphere, side of ss Wada administration, and the vessels selected for each case. Lesion indicates the side of the lesion based on imaging, Dominant indicates the language-dominant hemisphere, and Selected vessels lists the number of MCA branches and any concomitantly selected ACA or PCA branches.

MCA(x) indicates the number of MCA branches selectively injected. “/” separates vessels injected on the left and right hemispheres.

### 3.2 Super selective Wada test-based functional mapping

The positivity rates for functional tasks according to super-selected MCA branches are summarized in Table 2. In the dominant hemisphere, language tasks showed high positivity in the MCA superior trunk, with comparable rates across repetition, naming, and reading, whereas the inferior trunk showed higher positivity for repetition and reading than for naming. For calculation, positive responses were observed in 9 of 15 patients (60.0%), more frequently involving the MCA superior than the inferior trunk. Line bisection demonstrated similar positivity rates in the dominant and non-dominant hemispheres, with relatively frequent involvement of the intermediate trunk. Motor weakness, assessed by arm drift and impaired handgrip, was frequently observed in the anterior cerebral artery (ACA) and MCA superior trunk, and was also noted in approximately 30% of inferior trunk injections.

**Table 2:** Positivity rates for motor, language, calculation, and spatial neglect tasks following super-selective injection into each middle cerebral artery (MCA) branch.

Task	Hemisphere	MCA superior	MCA inferior	MCA others
Verbal				
Repetition	Dominant	12/16 (75.0%)	15/16 (93.8%)	3/4 (75.0%)
Naming	Dominant	11/16 (68.8%)	9/16 (56.3%)	2/4 (50.0%)
Reading	Dominant	12/16 (75.0%)	15/16 (93.8%)	2/4 (50.0%)
Calculation	Dominant	8/15 (53.3%)	2/15 (13.3%)	1/4 (25.0%)
Hemispatial neglect	Dominant	6/13 (46.2%)	4/11 (36.4%)	2/3 (66.7%)
	Non-dominant	4/9 (44.4%)	3/10 (30.0%)	1/1 (100%)
Upper limb paralysis	Left	7/14 (50.0%)	5/14 (35.7%)	3/3 (100%)
	Right	4/11 (36.4%)	3/11 (27.3%)	2/2 (100%)

No formal hypothesis testing was performed. Values represent the number of patients with a positive response among those assessed for each task in each vascular territory. Denominators vary because not all tasks were performed in every patient; tasks were prioritized according to individual clinical relevance within the limited assessment time after propofol injection.

### 3.3 Clinical applications

We integrated super-selected vascular territories with SEEG-defined seizure-onset contacts and lesion location to support surgical decision-making. This information contributed to the indication for awake surgery and selection of intraoperative tasks in selected cases. Representative cases are presented below; case numbers correspond to Table 1.

Case 4: A right-handed man in his 40s was diagnosed with a left insular glioma and underwent ssWada followed by awake resection, resulting in a diagnosis of oligodendroglioma, NOS, CNS WHO grade 2. He postoperatively developed focal impaired awareness seizures (FIAS), which progressed to drug-resistant epilepsy despite adjustment of anti-seizure medicines (ASMs). Long-term video-EEG monitoring failed to clearly localize the SOZ, and SEEG was subsequently performed targeting the perilesional region and mesial temporal structures. Four SOZ contacts were identified. Fusion of post-SEEG CT, postoperative 3D FLAIR MRI, ssWada, and CTP demonstrated that one SOZ contact was within the ACA territory and the remaining two were within the MCA inferior trunk territory (Figure 1). No deficits were elicited during ACA injection, whereas all language tasks were impaired during inferior trunk injection. Because resection would have required awake surgery with uncertain feasibility of removing all SOZ, the patient elected continued medical management.

**Figure 1:**
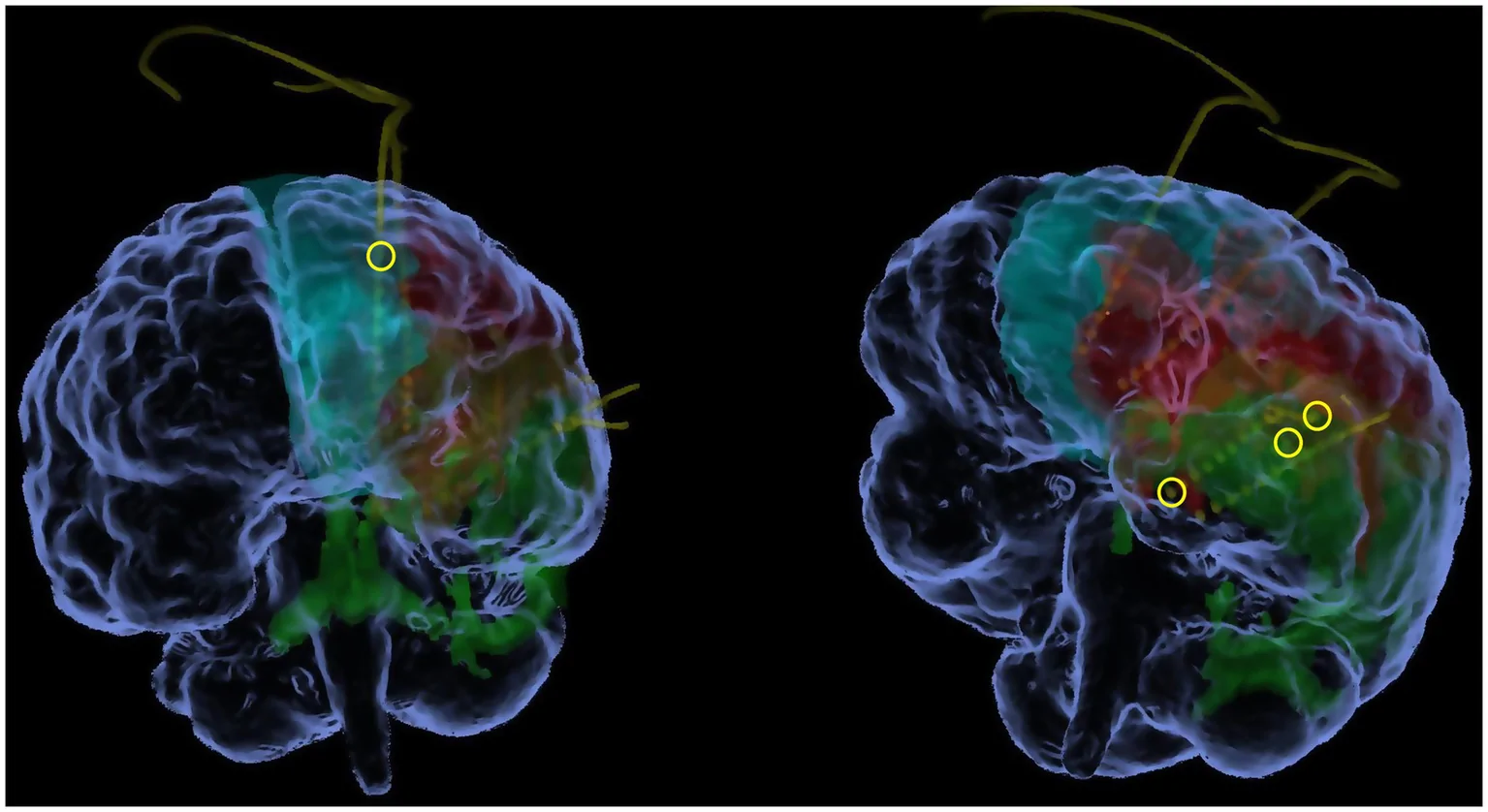
Fusion of super-selective Wada test and perfusion territories in a drug-resistant epilepsy patient (Case 4). Blue indicates the anterior cerebral artery (ACA), red indicates the middle cerebral artery (MCA) superior trunk, and green indicates the MCA inferior trunk perfusion territories. Yellow circles indicate SEEG-identified seizure onset zone (SOZ) contacts. Among the SOZ contacts, two were located within the MCA inferior trunk territory (green), one within the ACA territory (blue), and one in the medial temporal lobe outside both ACA and MCA territories. This fusion image clearly visualizes the relationship between the SOZ and perfusion territories, which informed treatment planning.

Case 16: A right-handed woman in her 70s with a CCM located at the right middle temporal gyrus developed progressive autonomic seizures despite ASM therapy. She worked as a sign-language interpreter and strongly requested preservation of sign-language ability. To evaluate functional involvement, ssWada including a sign-language task was performed. During selective injection of the right MCA inferior trunk, she showed task delay, and the lesion was located within the same vascular territory. Awake resection was therefore performed. The CCM was located on the cortical surface of the middle temporal gyrus, within the perfusion territory of the MCA inferior trunk. During awake resection, no impairment of sign-language performance was observed during cortical or subcortical stimulation around the lesion. Postoperatively, she has remained seizure-free for over 1 year (Engel class I) and continues her professional work as an interpreter.

Case 22: A right-handed woman in her 30s had undergone left parietal meningioma resection in adolescence and subsequently developed intermittent FIAS with nausea. After a prolonged period of stable control on ASM therapy, autonomic symptoms gradually worsened. Long-term video-EEG monitoring captured frequent nausea episodes without clear localization of the SOZ, and SEEG was therefore performed targeting the resection cavity and mesial temporal structures. One contact located posterolateral to the resection cavity was identified as the SOZ. ssWada demonstrated left-hemisphere language dominance. Three MCA branches were tested; injection into the inferior trunk induced language disturbance. Fusion of SEEG, postoperative MRI, and ssWada/CTP demonstrated that the SOZ lay within the inferior trunk territory (Figure 2). Awake resection was therefore planned. During awake surgery, surgical manipulation with bipolar forceps or a suction device at a site approximately 10 mm from the SOZ contact, as measured using neuronavigation, reproduced the patient’s habitual nausea. As resection proceeded around this region, the habitual symptom was no longer elicited. During subsequent additional resection around the SOZ, subcortical stimulation induced aphasia, consistent with the language disturbance observed during ssWada. After resection, the habitual symptoms were no longer observed. Postoperatively, she experienced transient aphasia and has remained free of preoperative weekly seizures.

**Figure 2:**
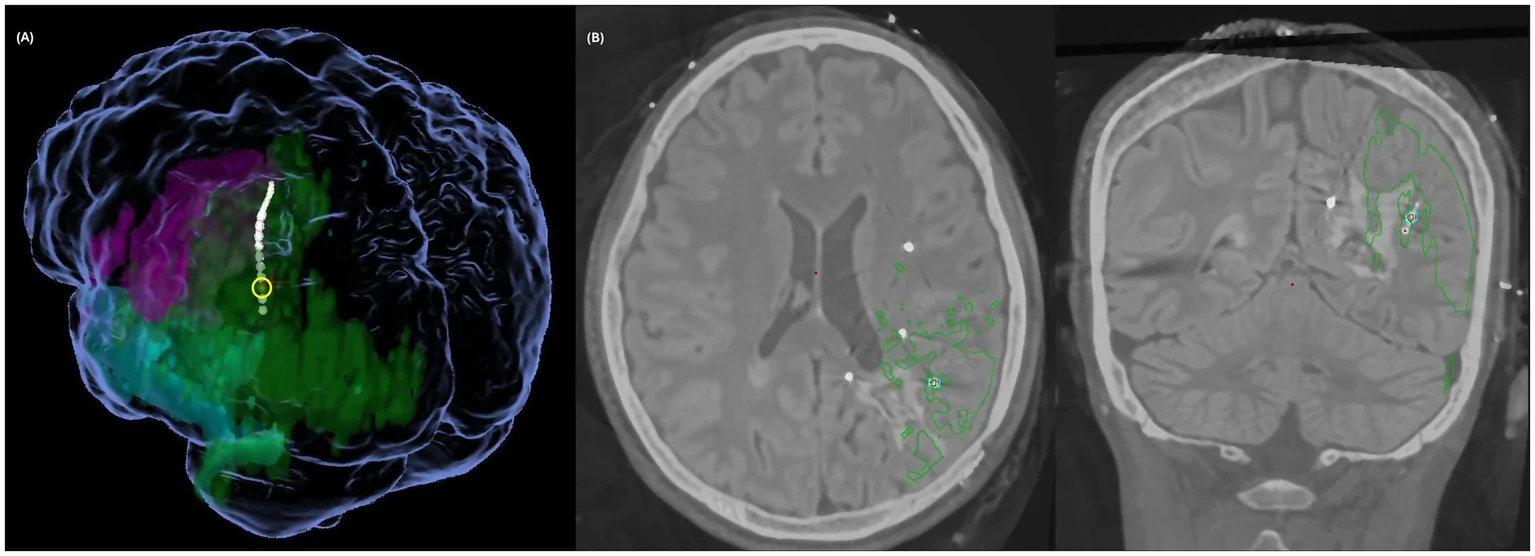
Fusion of super-selective Wada test and perfusion territories in a drug-resistant epilepsy patient (Case 22). **(A)** 3D FLAIR fat-suppressed MRI overlaid with MCA trunk perfusion territories: red indicates the middle cerebral artery (MCA) superior trunk; green indicates the MCA inferior trunk; blue indicates the MCA temporal artery. Yellow circles indicate SEEG-identified seizure onset zone (SOZ) contacts, which are located within the MCA inferior trunk territory (green). **(B)** Axial and coronal views showing the MCA inferior trunk perfusion territory (green lines). SOZ contacts located lateral to the resection cavity (cyan circles) are included within the inferior trunk territory.

As demonstrated in these cases, ssWada combined with CTP enables clear visualization of vascular territories corresponding to lesions or SEEG-defined SOZs. The fused images can be imported into the neuronavigation system, allowing direct intraoperative clinical application.

## 4 Discussion

In this study, we visualized functional mapping using the Wada test by combining ssWada with CTP. To our knowledge, no prior reports have described imaging-based Wada mapping. This approach enables localization of lesions or SEEG-identified SOZ contacts within specific functional territories before resection.

### 4.1 ssWada-based functional mapping across cerebral perfusion territories

Table 2 summarizes the symptoms elicited across MCA perfusion territories for the various tasks, with no statistically significant differences in positive rates. The MCA perfuses a broad cortical territory, and its branching patterns, including bifurcations and trifurcations, result in substantial inter-individual variation in perfusion regions (Kahilogullari et al., 2012).

For language tasks, the positivity rate in the superior trunk was approximately 70% across all tasks, suggesting that the superior trunk alone may not cover all language-related regions. About 20% of cases show language dominance in the superior trunk, whereas the inferior trunk is dominant in roughly half of cases (Kahilogullari et al., 2012; Türe et al., 2000). This is consistent with our finding that repetition and reading tasks showed high positivity rates in the inferior trunk. In contrast, naming tasks yielded positivity rates below 60%, suggesting task-specific differences in language processing. These findings are consistent with prior fMRI studies showing distinct involvement of the visual word form area and lateral occipital complex in reading and naming tasks (Cohen and Dehaene, 2004; Neudorf et al., 2022).

For calculation tasks, the superior trunk showed the highest positivity rate (>50%). fMRI studies have implicated the superior parietal lobule and other parietal regions in arithmetic processing (Rickard et al., 2000), while awake surgery reports have demonstrated calculation-related deficits following stimulation of the inferior parietal lobule and other parietal and frontal regions (Roux et al., 2009). Our findings showed that positive responses to simple calculations were observed relatively frequently in lateral frontoparietal regions. Line bisection tests yielded positive findings in both dominant and non-dominant hemispheres. Although hemispatial neglect is classically associated with non-dominant hemisphere lesions (Corbetta and Shulman, 2011), positive findings were also observed in the dominant hemisphere in our cohort.

### 4.2 Conventional functional mapping and the position of ssWada

Noninvasive modalities such as fMRI and MEG allow whole-brain functional evaluation but have limited specificity in perilesional regions. Blood oxygen level dependent signals can be distorted by lesion-related artifacts or disrupted neurovascular coupling, potentially failing to accurately reflect underlying neural activity (Colasurdo et al., 2023; Wang et al., 2012). Reports examining the relationship between fMRI findings and SOZ contacts or cerebral CCM remain limited, and even when combined with Diffusion Tensor Imaging tractography, accurate functional localization around pathological lesions remains challenging (Leone et al., 2025).

Cortical stimulation mapping during awake craniotomy is highly reliable but carries procedure-specific risks (Stevanovic et al., 2016; Eseonu et al., 2017; Spena et al., 2017). Awake surgery may be unsuitable for some patients, necessitating alternative neurophysiological mapping methods (Rossini et al., 2015; Spampinato et al., 2023; Toleikis et al., 2024), including cortico-cortical evoked potentials, which can substitute for language function monitoring (Matsumoto et al., 2004; Yamao et al., 2017; Mariani et al., 2021). In epilepsy surgery, passive language mapping is performed after SEEG implantation, whereas direct functional mapping is typically achieved with subdural cortical stimulation (Mitsuhashi et al., 2024; Kawase et al., 2025).

The conventional Wada test has long been established as a reliable method for assessing hemispheric dominance, and reports on ssWada have recently increased (Kakinuma et al., 2022; Kakinuma et al., 2024; Fujii et al., 2011). ssWada enables preoperative functional assessment through pharmacological inactivation based on cerebral perfusion territories. To our knowledge, relating functional findings from ssWada to patient-specific surgical anatomy for preoperative functional mapping remains challenging. Combining ssWada with CTP may facilitate visualization of the relevant perfusion territory and its spatial relationship to lesions and SEEG-defined SOZs, providing additional information for preoperative surgical planning.

### 4.3 Clinical utility

Preoperative images generated with ssWada and CTP provide visualization of which perfusion territories contain lesions or SEEG-defined SOZ contacts. SEEG is less invasive than conventional subdural electrodes and is associated with lower complication rates (Bandopadhay et al., 2025), while providing broader coverage including deep structures (Wu et al., 2024) and enabling safe electrical stimulation–based functional neuroanatomy identification (Grande et al., 2020). As SEEG is increasingly becoming the predominant modality for intracranial EEG, this approach is highly valuable for delineating the functional territories relevant to surgical planning.

When combined with CTP, ssWada provides imaging-based functional mapping by linking cerebral perfusion territories with SEEG-defined epileptogenic networks. Importantly, the clinical relevance of a functionally responsive territory depends not only on the function associated with that territory, but also on its spatial relationship to the individual patient’s lesion and SOZ contacts. When a functionally responsive territory overlaps or lies adjacent to the planned surgical target, this information may be relevant to postoperative functional risk assessment, whereas spatial separation from the target may support a different preoperative strategy. In SEEG-guided surgery, evaluating which ACA, MCA, or PCA subterritories contain SOZ contacts is clinically valuable for preoperative risk assessment of neurological deficits and for determining the necessity of awake surgery. These functional maps can also be integrated into neuronavigation systems for intraoperative reference. This approach is applicable not only to epilepsy but also to brain tumors or vascular lesions requiring SEEG, supporting function-preserving surgical planning.

### 4.4 Limitation

This study has some limitations. First, super-selective vessels were chosen according to lesion location, and the ACA, MCA, and PCA were not uniformly assessed. In addition, MCA branching patterns and perfusion territories vary substantially among individuals. Therefore, such descriptive associations between vascular territories and function are insufficient for generalization. Quantitative analysis of the relationship between perfusion territories and function remains a subject for future investigation. Substantial interindividual variability in cerebrovascular anatomy, including MCA trifurcation and heterogeneous perfusion territories, limits the validity of assumptions based on generalized anatomical models. In this context, individualized perfusion-based evaluation with CTP may provide a more accurate approach for identifying functional territories and guiding surgical planning. Second, assessment tasks were limited to repetition, naming, reading, single-digit calculation, and line bisection; other cognitive domains were not evaluated. Further optimization of task selection within a limited testing time is warranted. Third, the CTP-derived perfusion territory was used as a surrogate for the distribution of propofol. Although the two agents may differ in their injection dynamics, CTP was performed immediately before propofol administration with the microcatheter maintained in the same position. Thus, the CTP-derived territory was considered a reasonable approximation of the propofol perfusion territory, although exact correspondence cannot be guaranteed. Fourth, multiple assessments could be obtained from the same patient, resulting in within-patient clustering. Formal statistical comparisons were therefore not performed, and the vascular-territory findings are presented descriptively.

## 5 Conclusion

By integrating the super-selective Wada test with CTP, we visualized task-related functional changes within individual arterial perfusion territories. This represents an imaging-based application of the Wada test that has not been previously described. This approach provides clinically valuable information for individualized presurgical planning, including assessment of functional risk and determination of the necessity for awake craniotomy.

## Data Availability

The original contributions presented in the study are included in the article/supplementary material, further inquiries can be directed to the corresponding author.
